# Natural Killer Cell Education Is Associated With a Distinct Glycolytic Profile

**DOI:** 10.3389/fimmu.2018.03020

**Published:** 2018-12-19

**Authors:** Caroline Pfeifer, Andrew J. Highton, Sven Peine, Jürgen Sauter, Alexander H. Schmidt, Madeleine J. Bunders, Marcus Altfeld, Christian Körner

**Affiliations:** ^1^Research Department Virus Immunology, Heinrich Pette Institute, Leibniz Institute for Experimental Virology, Hamburg, Germany; ^2^Institute for Transfusion Medicine, University Medical Center Hamburg-Eppendorf, Hamburg, Germany; ^3^DKMS Gemeinnützige GmbH, Tübingen, Germany; ^4^DKMS Life Science Lab, Dresden, Germany; ^5^Department of Experimental Immunology and the Emma Children's Hospital, Amsterdam University Medical Center, University of Amsterdam, Amsterdam, Netherlands; ^6^Institute of Immunology, University Medical Center Hamburg-Eppendorf, Hamburg, Germany

**Keywords:** HLA class I, metabolism, NK-cell education, glycolysis, Glut1, killer-cell immunoglobulin-like receptor (KIR), cytotoxicity

## Abstract

NK cells expressing self-inhibitory receptors display increased functionality compared to NK cells lacking those receptors. The acquisition of functional competence in these particular NK-cell subsets is termed education. Little is known about the underlying mechanisms that lead to the functional differences between educated and uneducated NK cells. An increasing number of studies suggest that cellular metabolism is a determinant of immune cell functions. Thus, alterations in cellular metabolic pathways may play a role in the process of NK-cell education. Here, we compared the glycolytic profile of educated and uneducated primary human NK cells. KIR-educated NK cells showed significantly increased expression levels of the glucose transporter Glut1 in comparison to NKG2A-educated or uneducated NK cells with and without exposure to target cells. Subsequently, the metabolic profile of NK-cell subsets was determined using a Seahorse XF Analyzer. Educated NK cells displayed significantly higher rates of cellular glycolysis than uneducated NK cells even in a resting state. Our results indicate that educated and uneducated NK cells reside in different metabolic states prior to activation. These differences in the ability to utilize glucose may represent an underlying mechanism for the superior functionality of educated NK cells expressing self-inhibitory receptors.

## Introduction

Natural killer (NK) cells are a subset of lymphocytes that play a central role in the innate immune defense against tumors and viral infections (1). NK cells exert cytotoxicity toward aberrant target cells through release of cytolytic proteins, such as perforin and granzymes, and produce pro-inflammatory cytokines, such as IFN-γ and TNF-α. In addition to their role as cytotoxic effector cells, NK cells also function as immune regulators, influencing the maturation and activation of dendritic cells, macrophages, and T cells (2). NK-cell function is highly dependent upon the integration of signals derived from a variety of activating and inhibitory receptors expressed on the cell surface (3).

Activating NK-cell receptors mainly recognize cellular stress ligands that are upregulated on transformed or virus-infected host cells. In contrast, several inhibitory receptors are able to recognize human leucocyte antigen (HLA) class I molecules, which are ubiquitously expressed on the surface of nucleated cells. In humans, HLA class I-specific inhibitory receptors include the germline encoded killer-cell immunoglobulin-like receptor (KIR) family and the lectin-like CD94-NKG2A heterodimer (4–6). The 14 members of the KIR family are predominantly expressed on mature NK cells (7) and possess different specificities for HLA class I molecules (8). The inhibitory receptors KIR2DL1, KIR2DL2, KIR2DL3 recognize HLA-C molecules with different affinities (9, 10). Based on sequence polymorphism at amino acid position 80, HLA-C molecules can be subdivided into two principal groups: HLA-C group 2 (Lys^80^) and HLA-C group 1 (Asn^80^) (11, 12). KIR2DL1 binds exclusively to HLA-C group 2 allotypes, whereas KIR2DL3 recognizes HLA-C group 1 molecules. KIR2DL2, separating as a distinct allele from KIR2DL3, shows affinities for both groups (13). In contrast, KIR3DL1 recognizes HLA-A and HLA-B molecules expressing the Bw4 epitope (14, 15). Finally, the non-classical HLA-E molecule serves as a ligand for CD94/NKG2A (16). Subsequent interaction of inhibitory NK cell receptors with their HLA class I ligand on healthy host cells prevents NK cell activation and provides self-tolerance (17). Virus-infected or malignant host cells may downregulate HLA class I molecules to evade recognition by T cells (18). NK cells, however, are able to sense the absence of self-HLA class I molecules through the loss of inhibitory signals leading to NK-cell activation and target cell killing (“missing self”) (19, 20).

The NK-cell pool of every individual is characterized by a high diversity, determined by the receptors expressed on a single-cell level. A given NK cell can (i) express inhibitory receptors that recognize host HLA class I molecules leading to self-tolerance (self-inhibitory), (ii) express inhibitory receptors that fail to interact with host HLA class I molecules in individuals lacking cognate HLA class I ligands for these receptors, or (iii) lack inhibitory receptors all together. Several studies have shown that certain KIRs in combination with the cognate HLA class I-haplotype affect NK-cell effector potency (21). NK cells that lack self-inhibitory receptors have been shown to be hyporesponsive after exposure to MHC class I devoid target cells (20, 22–25). Consequently, the observed differences in NK-cell responsiveness are determined by the inhibitory receptor repertoire expressed by each NK cell and the corresponding HLA class I haplotype of the host. The process that regulates the level of NK-cell responsiveness is defined as NK-cell education (22, 26).

Several models of NK-cell education hypothesize how NK-cell responsiveness is achieved, but the molecular mechanisms underlying NK-cell education are not well understood. Recent studies in the field of immunometabolism have revealed that cellular metabolism is able to shape immune cell effector functions (27) which suggests that NK-cell metabolism might play a role in education as well. Mature naive T cells undergo metabolic remodeling following immune activation through T cell receptor ligation. Activated T cells switch from a metabolically quiescent state to a program in which aerobic glycolysis is upregulated (28). As cells proliferate, expression of nutrient transporters is induced on the cell surface (29). It has been shown that the glucose transporter Glut1 is critical for T cell growth and proliferation and that its knockdown results in suppressed glycolytic function and impaired proliferation in human T cells (30). Aligned with these findings, recent reports have shown that metabolic processes are essential for NK-cell effector functions (31). By limiting the rate of glycolysis, NK-cell effector functions, such as IFN-γ production and granzyme B expression, are inhibited (32). Given the link between distinct metabolic profiles and immune effector functions, we assessed the metabolic profiles of primary human NK cells in the setting of NK-cell education.

## Methods

### Cell Lines

The MHC class I devoid cell lines 721.221 (33) and K-562 (34) were used as target cells to assess NK-cell function. Cells were cultivated in complete medium (RPMI 1640 Medium, Thermo Fisher Scientific, Waltham, MA, USA) supplemented with 10% (v/v) heat-inactivated fetal bovine serum (FBS, Biochrome, Berlin, Germany), 100 units/ml penicillin and 0.1 mg/ml streptomycin (Sigma-Aldrich, St. Louis, Missouri, USA).

### Sample Acquisition and Processing

Peripheral blood samples were obtained from 45 randomly selected healthy blood donors recruited at the Institute for Transfusion Medicine, University medical center Hamburg-Eppendorf, Hamburg, Germany. For this study, residual amounts of anonymized peripheral blood samples were used which were routinely taken from healthy blood donors and would have been discarded otherwise. All blood donors gave their general written consent to use their blood samples for scientific studies in an anonymized form. The anonymized use of this material complies with a vote by the ethics committee of the German Medical Association. Furthermore, peripheral blood samples were obtained from six additional healthy blood donors recruited at the University medical center Hamburg-Eppendorf, Hamburg, Germany. These donors provided written informed consent and studies were approved by the ethical committee of the Ärztekammer Hamburg. Peripheral blood mononuclear cells (PBMCs) were isolated by density-gradient centrifugation within 2 h of sample collection, washed, and subsequently resuspended in cell culture medium (RPMI 1640 Medium, Thermo Fisher Scientific, Waltham, MA, USA) supplemented with 10% (v/v) heat-inactivated FBS (Biochrome, Berlin, Germany).

### Enrichment of NK Cells

Primary NK cells were enriched from PBMCs through an immunomagnetic negative selection strategy (EasySep Human NK cell Isolation Kit, Stemcell Technologies, Vancouver, Canada) according to the manufacturer's protocol. Purity of the enriched cell populations was determined by flow cytometry using fluorochrome-conjugated antibodies against CD3, CD14, CD16, CD19, and CD56. Isolated NK cells were resuspended in complete medium at a density of 3 × 10^6^ cells/ml and cultured overnight in the presence of 5 ng/ml recombinant human IL-15 (PeproTech GmbH, Hamburg, Germany) at 37°C, 5% (v/v) CO_2_.

### Antibodies and Flow Cytometry

Multi-parameter flow cytometry was used for phenotypical and functional characterization of NK cells as well as for cell sorting of NK-cell subsets according to their expression of KIR2DL1, KIR2DL2/L3, KIR3DL1, and NKG2A. Of note, the anti-KIR2DL1 antibody that was used in this study has been shown to recognize KIR2DS5 (35). In addition, the anti-KIR2DL2/L3 antibody can recognize KIR2DS2. The cross-reactivity of these antibodies was considered negligible. Cells were acquired using a BD LSRFortessa flow cytometer (BD Biosciences, Franklin Lakes, NJ, USA). Flow cytometric sorting of NK-cell subsets was performed using a BD FACSAria II SORP (BD Biosciences, Franklin Lakes, NJ, USA). Boolean and logic gating were used to determine NK-cell subsets that are either single positive for the respective inhibitory receptors, co-express certain combinations or lack the respective inhibitory receptors all together. The data was further analyzed using FlowJo 10.4.2 software (FlowJo, LLC, Ashland, OR, USA). A comprehensive list of all antibodies and reagents is provided in Supplementary Table 1. The corresponding gating strategy is displayed in Supplementary Figure 1.

### Assessment of NK-Cell Function

Levels of NK-cell activation were determined through expression of CD107a on the surface of NK cells as previously described (36). Enriched primary NK cells were cultured in the presence or absence of the MHC class I devoid target cell lines 721.221 or K-562 at an effector:target ratio of 1:2 for a total of 4 h in the presence of anti-human CD107a. Monensin (BD GolgiStop, BD Biosciences, Franklin Lakes, NJ, USA) was added 1 h after setup of the co-culture followed by additional 3 h of incubation at 37°C, 5% (v/v) CO_2_. Cells were then washed with PBS and stained for viability, expression of CD3, CD14, CD16, CD19, and CD56 as well as for the NK-cell receptors KIR2DL1, KIR2DL2/L3, KIR3DL1, and NKG2A. Glut1.RBD-GFP labeling was performed subsequently in RPMI 1640 medium supplemented with 10% (v/v) FBS, 0.09% (w/v) sodium azide (NaN_3_), and 1 mM EDTA for 30 min at 37°C. Cells were further washed with PBS, fixed in PBS supplemented with 2% (v/v) FBS, 0.09% (w/v) NaN_3_, 1 mM EDTA and 1% (w/v) paraformaldehyde (PFA) then acquired on a BD LSRFortessa flow cytometer. Educated NK-cell subsets were initially determined through differences in CD107a frequency using NK cells lacking the assessed inhibitory receptors as a reference for uneducated NK cells. Subsequently, the education status of NK-cell subsets was confirmed by HLA class I genotyping shown in Supplementary Table 2.

The effect of metabolic inhibitors on NK-cell degranulation was also tested. Prior to carrying out the degranulation (CD107a) assay, enriched NK cells were incubated with either 2.5 mM 2-Deoxy-D-glucose (2-DG) (Sigma-Aldrich) in complete medium, diluent control in complete medium, 2-DG in glucose free medium or in glucose free media alone for 2 h at 37°C, 5% CO_2_. The degranulation assay was subsequently carried out in the absence of 2-DG but matching the previous incubation with either complete or glucose free medium.

### Glucose Uptake Assay

The glucose uptake assay was performed as has been described previously (37). Enriched primary NK cells were incubated in glucose free medium (Thermo Fisher Scientific, Waltham, MA, USA) supplemented with 50 μM 2-NBDG (Biomol, Hamburg, Germany) for 30 min at 37°C, 5% (v/v) CO_2_. Subsequently, surface staining was carried out by incubating NK cells with LIVE/DEAD Fixable Blue dye and antibodies against CD3, CD14, CD16, CD19, and CD56 as well as for the NK cell receptors KIR2DL1, KIR2DL2/L3, KIR3DL1, and NKG2A. Cells were washed with PBS, fixed with 1% (v/v) PFA and then acquired on a BD LSRFortessa flow cytometer.

### Seahorse Assay

The Seahorse XFe96 Analyzer (Agilent Technologies, Santa Clara, CA, USA) was used to measure the glycolytic function of NK-cell subsets. Educated and uneducated NK cells were FACS-sorted on a BD FACSAria II SORP flow cytometer using enriched NK cells as primary material. Sorted NK cells were cultivated overnight in complete cell culture medium (RPMI 1640 Medium, Thermo Fisher Scientific, Waltham, MA, USA) supplemented with 10% (v/v) heat-inactivated FBS (Biochrome, Berlin, Germany) and 5 ng/ml recombinant human IL-15 (PeproTech, Hamburg, Germany). The following day 2 × 10^5^ to 1 × 10^6^ NK cells were resuspended in glucose-free assay medium prior to the analysis and then into a 96-well Seahorse XF cell culture microplate (Agilent Technologies, Santa Clara, CA, USA) and incubated for 30 min in a CO_2_-free incubator at 37°C. Sample replicates were used whenever sufficient cell numbers were available (max. triplicates). Subsequently, oxygen consumption rate (OCR) and extracellular acidification rate (ECAR) were measured in a Glycolysis Stress Test (Agilent Technologies, Santa Clara, CA, USA) using the manufacturer's protocol.

### Statistical Analysis

GraphPad Prism 7.04 (GraphPad Software, La Jolla, CA, USA) was used for statistical analyses and graphical display of the data. For multiple comparisons, Friedman test with subsequent Dunn's multiple comparisons test was used. Wilcoxon matched-pairs signed rank test with subsequent Bonferroni correction was used for two groups with paired values. All *p*–values shown are multiplicity adjusted. Adjusted *p*–values below 0.05 are considered statistically significant.

### Data Availability

Data storage is performed by the Heinrich Pette Institute on an internal server. Data are available upon request and can be shared after confirming that data will be used within the scope of the originally provided informed consent.

## Results

### Assessment of NK-Cell Function and Glycolytic Profile

**Figure 1:**
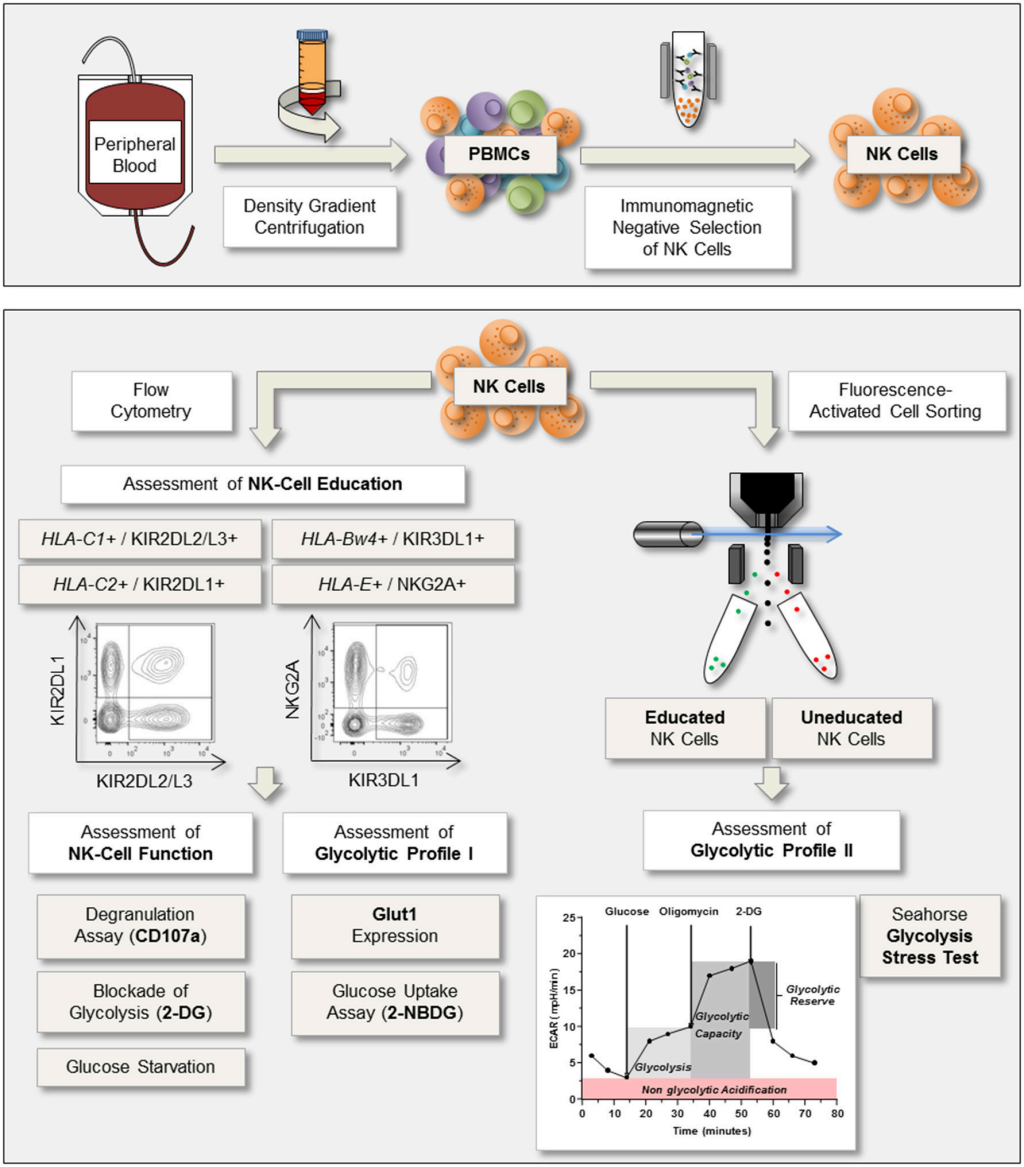
Experimental setup for the assessment of NK-cell function and glycolytic profile. **Upper panel**: Peripheral blood was processed through density gradient centrifugation to isolate peripheral blood mononuclear cells (PBMCs). Subsequently, NK cells were enriched via immunomagnetic negative selection. **Lower panel**: NK cells were analyzed via flow cytometry for expression of the inhibitory receptors KIR2DL1, KIR2DL2/L3, KIR3DL1, and NKG2A to identify NK cells positive for self-inhibitory receptors (educated). NK-cell function was determined by the expression of CD107a after co-culture with the MHC class I devoid target cell lines K-562 and 721.221. Simultaneously, expression of the glucose transporter Glut1 was examined to assess the glycolytic profile. In addition, glucose uptake assays were performed and the effect of the metabolic inhibitor 2-DG on NK-cell degranulation was investigated. Based on the expression of self-inhibitory receptors educated and uneducated NK cells were sorted the same day and rested overnight. The following day, a Glycolysis Stress Test was performed to calculate the rates for glycolysis, glycolytic capacity, and glycolytic reserve.

To investigate the glycolytic profiles of primary human NK cells in the context of NK-cell education, the workflow presented in Figure 1 was followed. First, function and education status of enriched NK cells were determined by the expression of CD107a after exposure to MHC class I deficient target cell lines (721.221 and K-562 cells). Simultaneously, expression of the glucose transporter Glut1 was determined. In addition, the glycolytic profile of FACS-sorted educated and uneducated NK cells was determined using the Seahorse XF Glycolysis Stress Test.

### Educated NK Cells Exhibit Enhanced Degranulation Capacity Compared to Uneducated NK Cells

**Figure 2:**
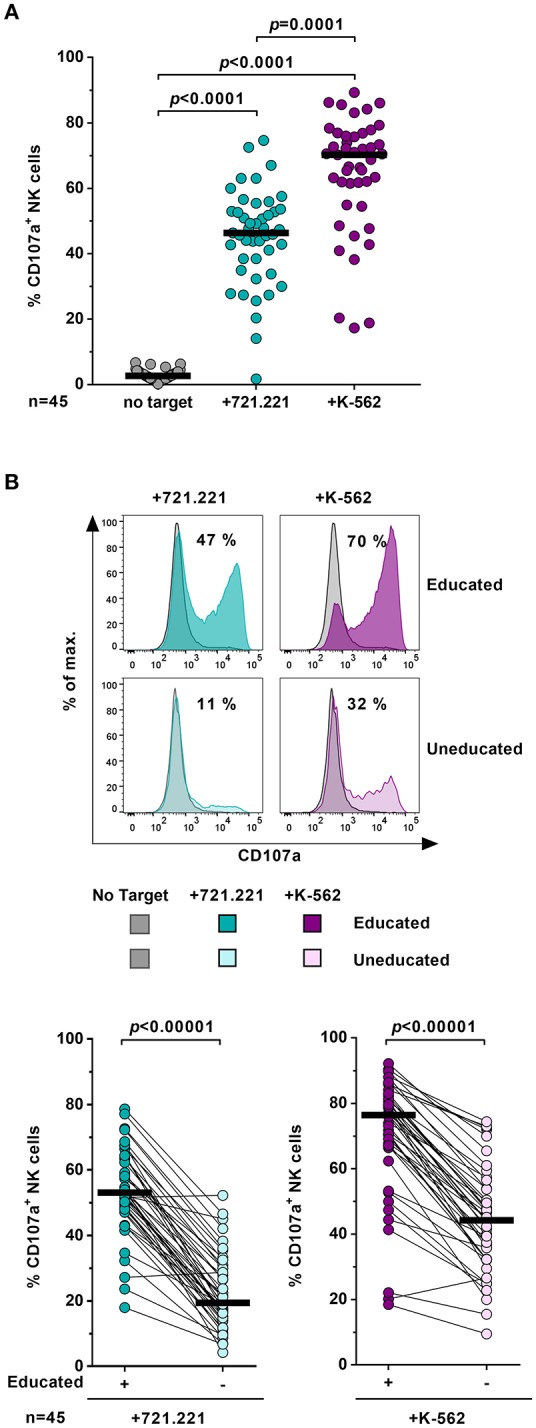
Education of primary NK cells. Flow cytometric assessment of NK-cell function after exposure to various target cells. Enriched primary NK cells from healthy donors (*n* = 45) were cultured either in the absence (gray) or presence of 721.221 cells (cyan) or K-562 cells (purple). **(A)** Proportion of CD107a^+^ bulk NK cells. Statistical analysis: Friedman test, Dunn's multiple comparisons test. Black bars represent the median. **(B)** Upper panel: Representative histogram of CD107a expression of educated and uneducated NK cells after stimulation with target cells. Numbers indicate the percentage of CD107a^+^ cells after exposure to target cells. Lower panel: Comparison of CD107a expression frequency between educated and uneducated NK cells. Statistical analysis: Wilcoxon matched-pairs signed-rank test with subsequent Bonferroni correction. Black bars represent the median.

NK-cell function is tuned by the interaction between self-inhibitory receptors and their cognate HLA class I ligands. In order to assess NK-cell function in the context of host HLA class I haplotypes, levels of NK-cell degranulation were determined in the presence or absence of MHC class I devoid target cell lines 721.221 and K-562 (Figure 2). The frequency of CD107a^+^ cells was significantly increased on bulk NK cells in the presence of 721.221 cells (*p* < 0.0001) or K-562 cells (*p* < 0.0001) (Figure 2A). K-562 cells induced a stronger NK-cell response than 721.221 cells (*p* = 0.0001). After exposure to target cell lines, educated NK cells displayed a significantly higher percentage of CD107a^+^ NK cells than uneducated NK cells (*p* < 0.00001) (Figure 2B). Increased response rates were observed for all tested subsets expressing individual self-inhibitory receptors (Supplementary Figure 2). Here, we confirmed that the expression of self-inhibitory receptors was associated with increased functional competence of NK cells, enabling us to distinguish educated and uneducated NK-cell populations in the same donor for subsequent metabolic assessments.

### Educated NK Cells Show Differences in Glut1 Expression

**Figure 3:**
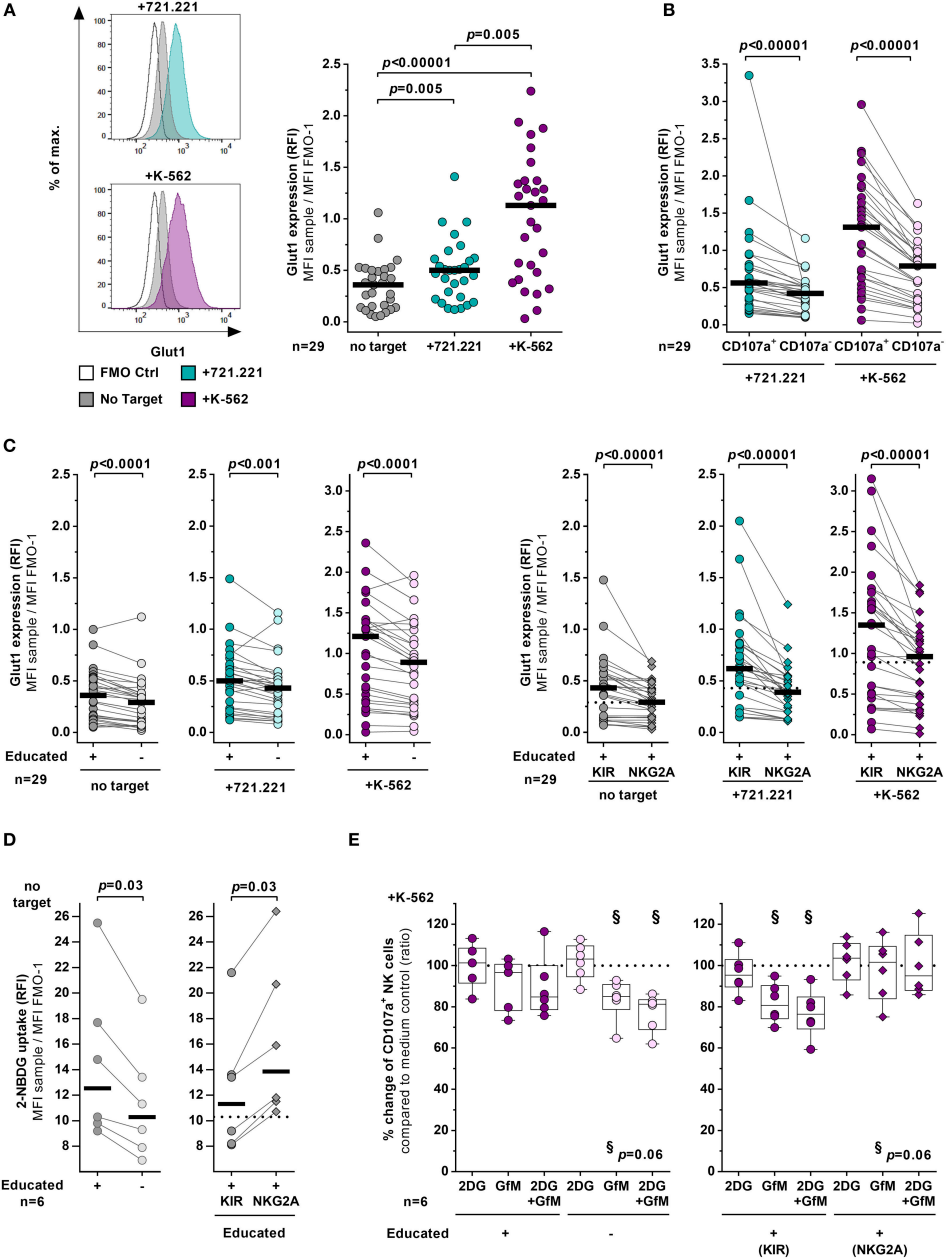
KIR-educated NK cells express higher levels of Glut1. **(A–C)** Flow cytometric assessment of the glucose transporter Glut1 expression on NK cells with and without exposure to various target cells. Enriched primary NK cells from healthy donors (*n* = 29) were cultured for 4 h either in absence (gray) or presence of 721.221 cells (cyan) or K-562 cells (purple). **(A)** Left panel: Representative histogram of Glut1 expression of bulk NK cells. Right panel: Relative fluorescence intensity (RFI) of Glut1 on bulk NK cells. Statistical analysis: Friedman test, Dunn's multiple comparisons test. Black bars represent the median. **(B)** Comparison of Glut1 expression (RFI) between CD107a^+^ and CD107a^−^ NK cells in presence of 721.221 cells (cyan) or K-562 cells (purple). Statistical analysis: Wilcoxon matched-pairs signed-rank test with subsequent Bonferroni correction. Black bars represent the median. **(C)** Comparison of Glut1 expression (RFI) between educated and uneducated NK cells (left panel) and between KIR- and NKG2A-educated NK cells (right panel). Statistical analysis: Wilcoxon matched pairs signed rank test with subsequent Bonferroni correction. Black bars represent the median. Dashed lines indicate the median of 2-NBDG fluorescence (RFI) of uneducated NK cells. **(D)** Flow cytometric assessment of glucose uptake by enriched primary NK cells using 2-NBDG (*n* = 6). Comparison of Glucose uptake between educated and uneducated NK cells (left panel) and between KIR- and NKG2A-educated NK cells (right panel). Statistical analysis: Wilcoxon matched pairs signed rank test. Black bars represent the median. Dashed lines indicate the median of 2-NBDG fluorescence (RFI) of uneducated NK cells. **(E)** Impact of metabolic inhibitors on NK cell degranulation. Enriched primary NK cells from healthy donors (*n* = 6) were pretreated with either 2-DG (2DG), glucose-free media (GfM), both or left in complete medium before cultured for 4 h either in absence or presence of K-562 cells. Scatter plots shows the relative change of CD107a^+^ cells in the treated samples compared to the medium control. Plots compare educated with uneducated NK cells (left panel) and KIR-educated with NKG2A-educated NK cells (right panel). Statistical analysis: Wilcoxon matched-pairs signed-rank test with subsequent Bonferroni correction. Boxes and whiskers indicate the median, 25/75% percentile and the minimum/maximum. Dashed line indicates 100%.

Expression of the glucose transporter Glut1 has been implicated in influencing effector functions of lymphocytes (30, 38). Therefore, expression levels of Glut1 in educated and uneducated NK cells were tested with and without cellular stimulation using MHC class I devoid cell lines (Figure 3). Bulk NK cells expressed significantly higher surface levels of Glut1 in the presence of 721.221 cells (*p* = 0.005) or K-562 cells (*p* < 0.00001) than NK cells in the absence of any target cell line (Figure 3A). Glut1 expression levels were more pronounced on bulk NK cells exposed to K-562 cells compared to NK cells co-cultured with 721.221 cells (*p* = 0.005) (Figure 3A, right panel). This is possibly due to the increased activation as demonstrated by higher CD107a expression in response to K-562 cells (Figure 2A). Indeed, when exposed to the respective target cell lines, CD107a^+^ NK cells exhibited higher expression of Glut1 on their cell surface than CD107a^−^ NK cells (*p* < 0.00001) (Figure 3B). Stratification of bulk NK cells into educated and uneducated cells revealed that educated NK cells express higher levels of Glut1 than uneducated NK cells after exposure to both tested target cell lines (721.221 cells: *p* < 0.001 and K-562 cells: *p* < 0.0001) (Figure 3C, left panel). Nevertheless, cellular stimulation of NK cells resulted in an upregulation of Glut1 in both educated and uneducated NK cell subsets (educated: 721.221 cells *p* < 0.05 and K-562 cells *p* < 0.0001, uneducated: 721.221 cells *p* = 0.02 and K-562 cells *p* < 0.0001, Supplementary Figure 3A). Of note, elevated surface expression levels of Glut1 were also observed in educated NK cells without activation (*p* < 0.0001) (Figure 3C, left panel). Further stratification of educated NK cells into KIR^+^ and NKG2A^+^ NK cells revealed significant differences in the expression of Glut1 between the two subsets (Figure 3C, right panel, Supplementary Figure 3B). At basal levels as well as after exposure to target cells educated KIR^+^ NK cells showed significantly higher expression of Glut1 compared to their NKG2A^+^ counterparts (*p* < 0.00001 each). Moreover, Glut1 expression levels did not differ between NKG2A^+^ NK cells and uneducated NK cells. To assess the general ability to absorb glucose irrespective of Glut1, a 2-NBDG uptake assay was performed (Figure 3D). As with Glut1, educated NK cells showed increased uptake of 2-NBDG compared to uneducated NK cells (*p* = 0.03, left panel), however, the ability to take up glucose was even further enhanced in NKG2A^+^ cells when compared to KIR-educated cells (*p* = 0.03, right panel). Finally, the potential effects of glycolysis blockade and glucose starvation on NK-cell cytotoxicity were investigated (Figure 3E). Treatment with the glycolysis inhibitor 2-DG alone had no effect on the ability of NK cells to degranulate after exposure to K-562 cells. In contrast, incubation with glucose-free media affected the frequency of CD107a^+^ NK cells in the uneducated subset and was even further pronounced in combination with 2-DG (*p* = 0.06, left panel). While the educated subset as a whole seemed to be unaffected by the culture conditions, stratification into KIR-educated and NKG2A-educated NK cells showed significant differences in the CD107a expression frequency. Glucose-free culture conditions had no effect on the percentage of CD107a^+^ NKG2A^+^ NK cells, whereas KIR-educated were impacted similarly compared to uneducated NK cells (*p* = 0.06, right panel).

Taken together, KIR-educated NK cells exhibited overall higher surface levels of Glut1 in comparison to NKG2A-educated and uneducated NK cells, which was observed after stimulation with target cell lines and of particular note in the absence of stimulation. In contrast, NKG2A^+^ NK cells showed an increased ability to take up 2-NBDG compared to KIR-educated and uneducated NK cells. Both of the latter subsets were also more susceptible to the lack of glucose in terms of their cytotoxic function compared to NKG2A^+^ NK cells.

### Educated and Uneducated NK Cells Display Different Glycolytic Profiles

**Figure 4:**
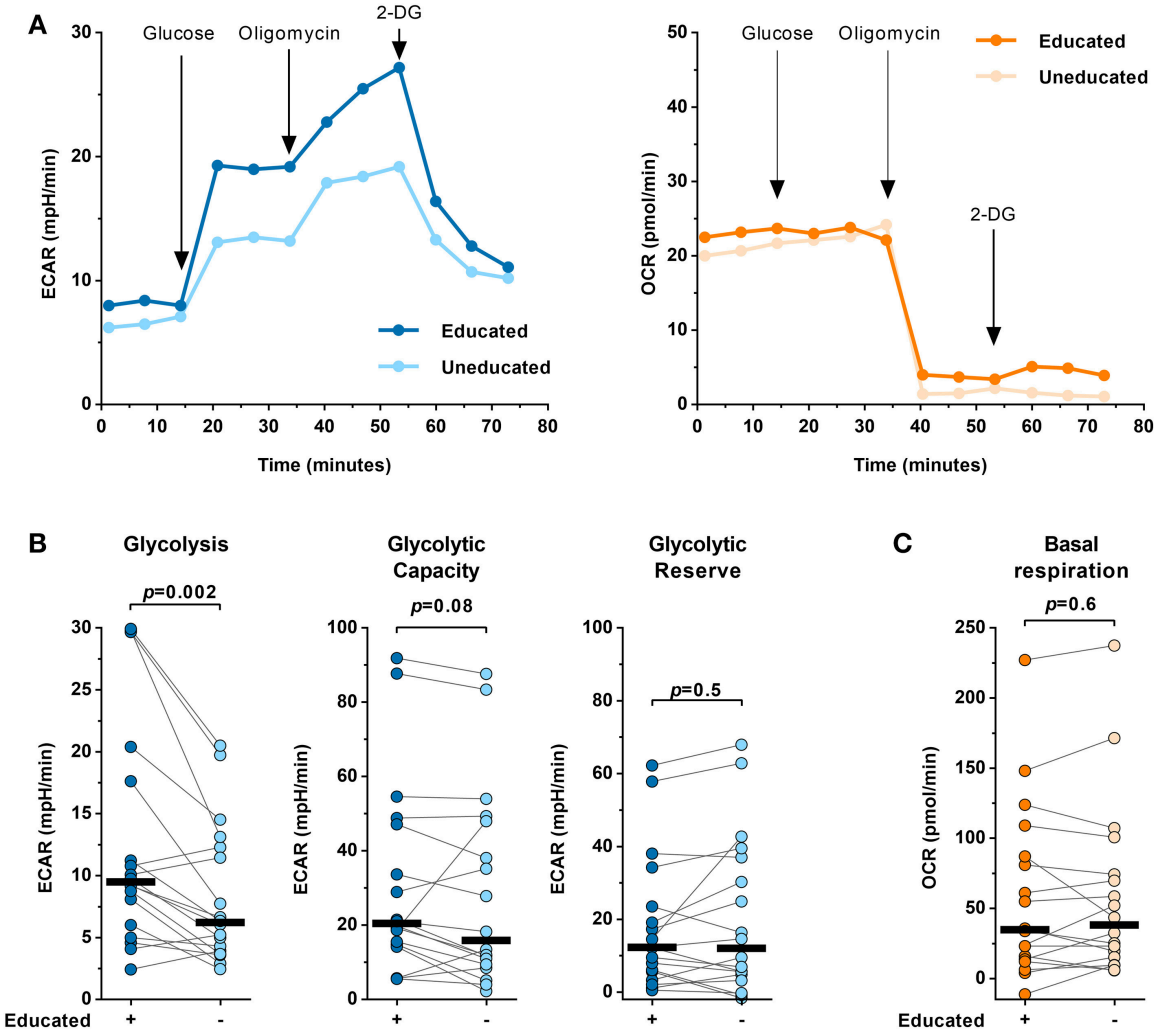
Educated and uneducated NK cells display different glycolytic profiles. The glycolytic profile of FACS-sorted educated and uneducated NK cells was determined in a Glycolysis Stress Test using the Seahorse XF extracellular flux analyzer (*n* = 18). **(A)** Representative ECAR (left panel) and OCR data right panel of educated and uneducated NK cells measured in a Glycolysis Stress Test from a single donor. **(B)** Comparison of glycolysis (left panel), glycolytic capacity (middle panel) and glycolytic reserve (right panel) between educated and uneducated NK cells calculated from ECAR data (*n* = 18). Statistical analysis: Wilcoxon matched pairs signed rank test with subsequent Bonferroni correction. Black bars represent the median. **(C)** Comparison of the basal respiration between educated and uneducated NK cells derived from OCR data (*n* = 18). Statistical analysis: Wilcoxon matched-pairs signed-rank test. Black bars represent the median.

Glycolytic metabolism is a key factor impacting lymphocyte function. To determine potential differences in the metabolic profile of educated and uneducated NK cells a Seahorse XF Glycolysis Stress Test was performed (Figure 4). The Seahorse XF Glycolysis Stress Test measures the glycolytic function of cells and provides information about several parameters of the glycolytic flux, including glycolysis, glycolytic capacity and glycolytic reserve. FACS-sorted educated and uneducated NK cells were tested using the Glycolysis Stress Test (Figure 4A). The Seahorse XF analyzer performs sequential measurements of the extracellular acidification rate (ECAR) and oxygen consumption rate (OCR) after the addition of glucose (fueling glycolysis), oligomycin [ATP synthase inhibitor blocking oxidative phosphorylation (OXPHOS)] and 2-DG (synthetic glucose analog inhibiting glycolysis). Our data showed that educated NK cells exhibited significantly higher ECAR values compared to uneducated NK cells (*p* = 0.002) (Figure 4B). When OXPHOS was interrupted, educated, and uneducated NK cells showed no differences in glycolytic capacity (*p* = 0.08). In addition, the glycolytic reserve did not differ between educated and uneducated NK cells (*p* = 0.5). Of note, no differences in the oxygen consumption rate between educated and uneducated NK cells were observed throughout the assay (data not shown) including basal respiration after the addition of glucose (Figure 4C, *p* = 0.6). Taken together, the results show that when OXPHOS is interrupted, educated and uneducated NK-cell subsets exhibited similar glycolytic capacity and reserve, whereas educated NK cells were able to utilize glucose significantly better (increased glycolysis) than uneducated NK cells.

## Discussion

The molecular mechanisms underlying the functional superiority of educated NK cells remain insufficiently understood. New insights from the field of immunometabolism show that immune cells can undergo metabolic reprogramming upon transition from a quiescent to an activated state (27). To assess whether metabolic reprogramming may be associated with the differences seen in the responsiveness of educated and uneducated NK cells we performed metabolic analyses of these distinct NK-cell populations. Educated NK cells showed higher expression of the glucose transporter Glut1 than uneducated NK cells on their cell surface. Interestingly, a difference was also seen within the educated NK-cell pool when comparing NK cells educated via NKG2A or KIRs. Metabolic analysis using Seahorse showed that educated NK cells exhibit a significantly increased rate of glycolysis compared to uneducated but no difference was seen in oxidative phosphorylation. Blockade of glycolysis with 2-DG in the absence of glucose partially reduced the functional output of both uneducated and KIR-educated NK cells but surprisingly not of NKG2A-educated NK cells. Taken together, our results indicate that educated and uneducated NK cells exhibit differences in their glycolytic metabolism and these differences may explain the large functional discrepancy between the two populations.

Immune cell activation is associated with an upregulation of nutrient transporters on the cell surface in order to ensure the utilization of available nutrients for the cellular energy production and assembly of biomolecules (39). Previously it has been shown that CD3/CD28 T cell receptor stimulation leads to an upregulation of the glucose transporter Glut1 on the surface of human T cells, which enables these cells to comply with increased energy demands (40, 41). In this study we demonstrated that educated NK cells have increased surface expression of Glut1 compared to uneducated NK cells with or without activation by target cell lines. It has previously been demonstrated that the functional and phenotypically divergent subsets of CD56^bright^ and CD56^dim^ NK cells exhibit different expression patterns of Glut1 in humans (38). CD56^bright^ NK cells are found to be more metabolically active and express higher Glut1 levels in a resting state compared to CD56^dim^ NK cells which express low Glut1 levels (38). This is a similar pattern to that seen in educated compared to uneducated NK cells suggesting that Glut1 may be upregulated due to increased demand for glucose by NK cells as is seen in T lymphocytes (30, 41, 42).

Although Glut1 expression was increased in educated compared to uneducated NK cells, Glut1 expression did go up in both populations following stimulation with target cells. This observation is in line with previous studies describing increased Glut1 expression on the cell surface of CD56^dim^ NK cells following cytokine stimulation (38). Overall, these data suggest that Glut1 upregulation may allow NK cells activated by cytokines or target cells to take up increased amounts of glucose from the surrounding medium to fuel cellular glycolysis and meet the increased energy demand for effector functions, such as cytokine production, degranulation or proliferation. Indeed, educated NK cells took up more of the glucose analog 2-NBDG compared to uneducated. Interestingly, uptake was increased in NKG2A educated NK cells compared to KIR educated cells despite Glut1 expression being higher in the KIR subset. This suggests that other transporters may also be important for glucose uptake in this population such as Glut3 and Glut4 that have also been found to be expressed on NK cells (43). Interestingly, Glut3 and Glut4 expression have also been seen in mouse and human CD4^+^ T cells in both a naïve and activated state with a suggested role in glucose uptake following activation (30, 44). Thus, higher Glut1 surface expression and perhaps expression of other glucose receptors may provide an advantage for educated NK cells that contributes to the superior effector function of educated NK cells over uneducated NK cells.

Activated immune cells are characterized by an increased uptake of glucose and elevated levels of glycolysis (27). We demonstrated that educated NK cells have increased glycolytic activity compared to uneducated NK cells using a Seahorse XF Glycolysis Stress Test and this is supported by similar work performed by Schafer et al. (45). It is possible that the increased expression of glucose transporter seen on educated NK cells is associated with a metabolic shift toward glycolysis. The increase in glycolysis matches data from T cells where utilization of glucose through aerobic glycolysis aids activated T cells in meeting their metabolic demands to fulfill their effector functions (46). Recent research has demonstrated that NK cells exhibit similar metabolic changes upon activation with cytokines demonstrating the importance of glycolysis for NK-cell effector function (32). In our work, enriched NK cells were rested with low-dose IL-15 overnight prior to analysis. We found that comparison of Glut1 expression and 2-NBDG uptake directly *ex vivo* was similar to that seen following overnight rest (data not shown) although we cannot eliminate the possibility that low-dose IL-15 enhances these differences.

Schafer et al. saw no difference in oxidative phosphorylation between educated and uneducated NK cell populations using Seahorse, matching our result (45). Although glucose fueled oxidative phosphorylation has been shown to play an important role in cytokine stimulated NK-cell function it is possible that this does not contribute to the differences seen in educated compared to uneducated NK cells (47). Amino acids and their transporters are also involved in the metabolic regulation of NK cells with Loftus et al. recently showing that glutamine transport controls cMyc expression and subsequently glycolysis in mouse NK cells (48). The mammalian target of rapamycin complex 1 has also been shown to be crucial in this process but may be activated through differing pathways depending on stimulus (32, 48, 49). Further work is required to find whether amino acids play a role in the functional differences seen between educated and uneducated NK cells and what role different signaling pathways play in this.

NK cells can be activated through different stimulatory signals, which leads to the activation of multiple signaling pathways. In this work we investigated the metabolic response of resting NK cells or those activated with K562 or 721.221 cell lines. It may be that in NK cells differing stimuli give rise to differing metabolic responses. Indeed, in human NK cells distinct cytokine combinations can lead to different metabolic changes (38). Cytokine stimulation in mouse NK cells has been shown to induce increased glucose uptake and glycolytic rates (32). Interestingly, inhibition of either glycolysis or OXPHOS can impair NK cell effector functions following cytokine stimulation (32, 38). In this work we found that blockade of glycolysis with 2-DG in the absence of glucose partially inhibited NK cell degranulation in uneducated NK cells but not in educated cells. Schafer et al. recently showed that combinatorial blockade of glycolysis and oxidative phosphorylation was required to effectively inhibit the cytotoxicity of educated NK cells while blockade of glycolysis with 2-DG alone was insufficient (45). Interestingly, when deconvoluting educated NK cells into KIR educated and NKG2A-educated populations we saw partial inhibition in the KIR^+^ population but not in the NKG2A^+^ population suggesting variable metabolic behavior even within the educated NK-cell pool.

The resistance of educated NK cell function to blockade of glycolysis suggests a role for these cells in disease settings with low availability of glucose such as cancer (50–52). In our work we found that NKG2A-educated NK cells were more resistant to blockade of glycolysis than their KIR-educated counterparts suggesting they may be ideally suited to function with low glucose availability. Interestingly, low glucose levels and hypoxic conditions have been shown to induce upregulation of the NKG2A ligand HLA-E on tumor cell lines but also in primary tissues (53, 54). This may confer a necessary survival advantage against NKG2A^+^ NK cells and assist in immune escape.

We have shown that the phenotypically and functionally different educated and uneducated NK-cell subsets can be further distinguished by their metabolic profile regarding glucose metabolism. Further studies are now required to elucidate the molecular pathways that link engagement of self-inhibitory receptors with subsequent changes in cellular metabolism in educated NK cells. Taken together, the present study demonstrates that educated NK cells reside in a distinct glycolytic state that may serve as an underlying mechanism for the increased functional potential of NK cells expressing self-inhibitory receptors.

## Author Contributions

MJB, MA, and CK designed the study. CK and CP designed the experiments. CP, AJH, and CK conducted the experiments. CP, AJH, and CK analyzed the data. CP, AJH, and CK wrote the manuscript. SP, JS, and AHS provided resources. MA provided funding. All authors critically reviewed the manuscript.

### Conflict of Interest Statement

The author JS was employed by the company DKMS gemeinnutzige GmbH, Tübingen, Germany. AHS was employed by company DKMS gemeinnutzige GmbH, Tübingen, Germany and DKMS Life Science Lab, Dresden, Germany. The remaining authors declare that the research was conducted in the absence of any commercial or financial relationships that could be construed as a potential conflict of interest.
