# Supplementary material for: Natural Killer Cell Education Is Associated With a Distinct Glycolytic Profile
Source: Front Immunol. 2018 Dec 19;9:3020. doi: 10.3389/fimmu.2018.03020 (PMC6305746; doi:10.3389/fimmu.2018.03020)
Supplement: Supplementary file 1 [file Data_Sheet_1.docx]

Supplementary Material

Natural Killer Cell Education Is Associated With

a Distinct Glycolytic Profile

Caroline Pfeifer^1^, Andrew J. Highton^1^, Sven Peine^2^, Jürgen Sauter^3^, Alexander H. Schmidt^3,4^, Madeleine J. Bunders^1,5^, Marcus Altfeld^1,6^, Christian Körner^1*^

^1^Research Department Virus Immunology, Heinrich Pette Institute, Leibniz Institute for Experimental Virology, Hamburg, Germany

^2^Institute for Transfusion Medicine, University Medical Center Hamburg-Eppendorf, Hamburg, Germany

^3^DKMS gemeinnützige GmbH, Tübingen, Germany

^4^DKMS Life Science Lab, Dresden, Germany

^5^Department of Experimental Immunology and the Emma Children's Hospital, Amsterdam University Medical Center, University of Amsterdam, Amsterdam, the Netherlands

^6^Institute of Immunology, University Medical Center Hamburg-Eppendorf, Hamburg, Germany

*** Correspondence:**
Christian Körner

[christian.koerner@leibniz-hpi.de](mailto:christian.koerner@leibniz-hpi.de)

# Supplementary Figures

## Supplementary Figure 1 | NK‑cell gating strategy


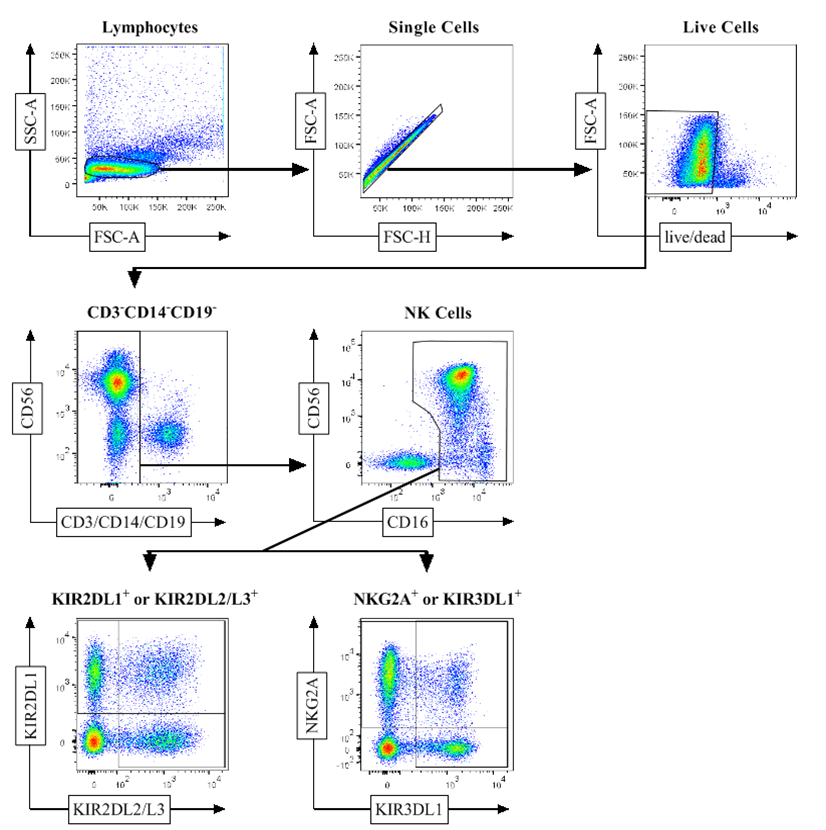


**Supplementary Figure 1 | NK-cell gating strategy**

Flow cytometric assessment of NK‑cell subsets. Lymphocytes were gated using forward and sideward scatter (FSC‑A/SSC‑A). Single cells were then identified by area and height of the forward scatter. Exclusion of dead cells was performed using the viability dye LIVE/DEAD Fixable Blue. Subsequently NK cells were identified by the exclusion of CD3^+^, CD14^+^, and CD19^+^ cells and the expression of either CD56 or CD16. NK‑cell subsets were further defined by the expression of the inhibitory receptors KIR2DL1, KIR2DL2/L3, KIR3DL1 and NKG2A.

## Supplementary Figure 2 | Increased levels of CD107a in educated NK-cell subsets


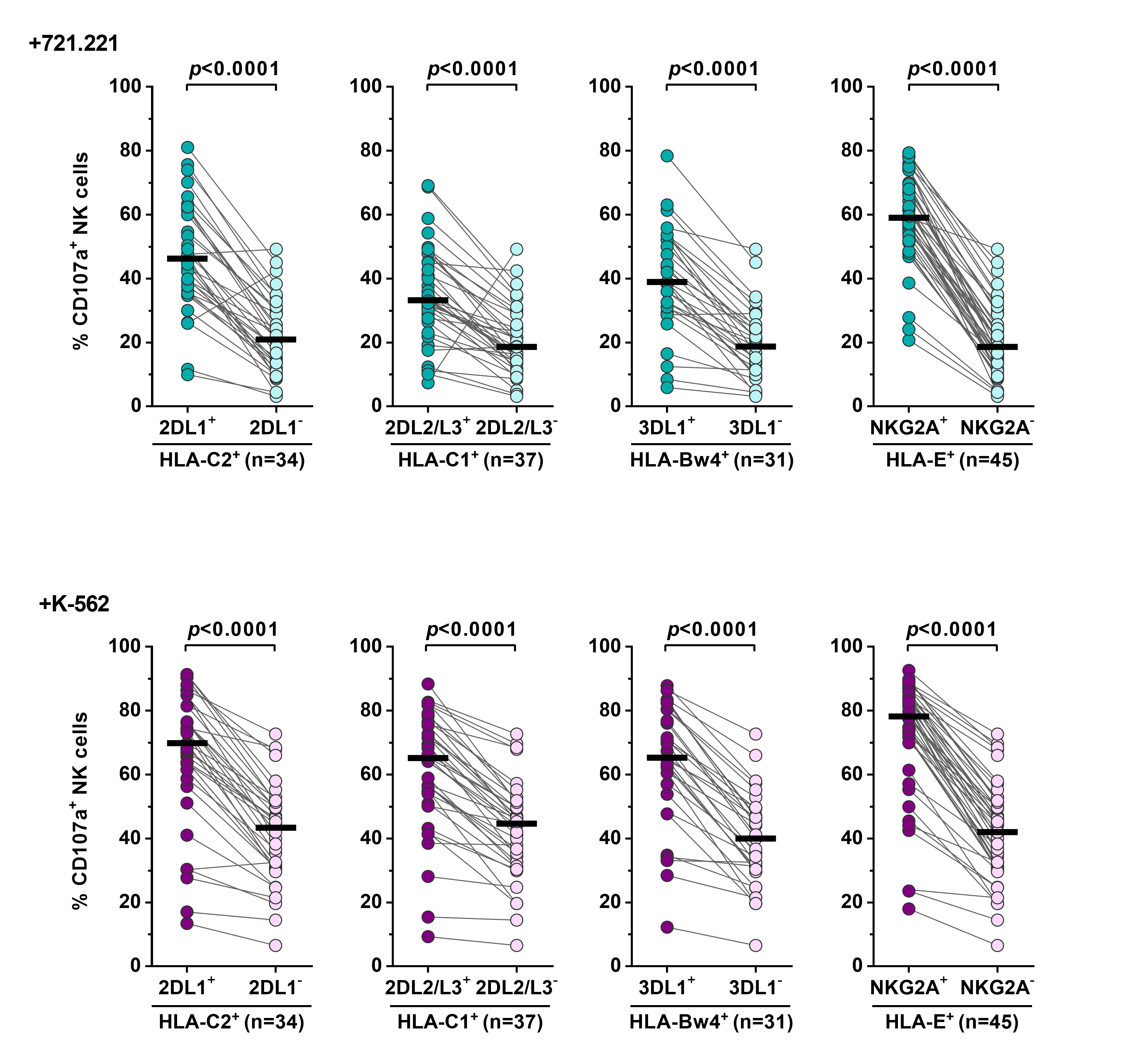


**Supplementary Figure 2 | Increased levels of CD107a in educated NK-cell subsets**

Flow cytometric assessment of NK‑cell function after exposure to various target cells. Enriched primary NK cells from healthy donors were cultured for 4 h in the presence of 721.221 cells (cyan) or K‑562 cells (purple). The figure shows the CD107a expression frequency of NK-cell subsets that are either single positive for one of the investigated self-inhibitory receptors (2DL1, 2DL2/L3, 3DL1 or NKG2A) or subsets that lack all of the investigated self-inhibitory receptors in donors that express the cognate HLA class I molecule (Upper panel: +721.221, lower panel: +K-562). Statistical analysis: Wilcoxon matched-pairs signed-rank test with subsequent Bonferroni correction. Black bars represent the median.

## Supplementary Figure 3 | Upregulation of Glut1 in educated and uneducated NK cell
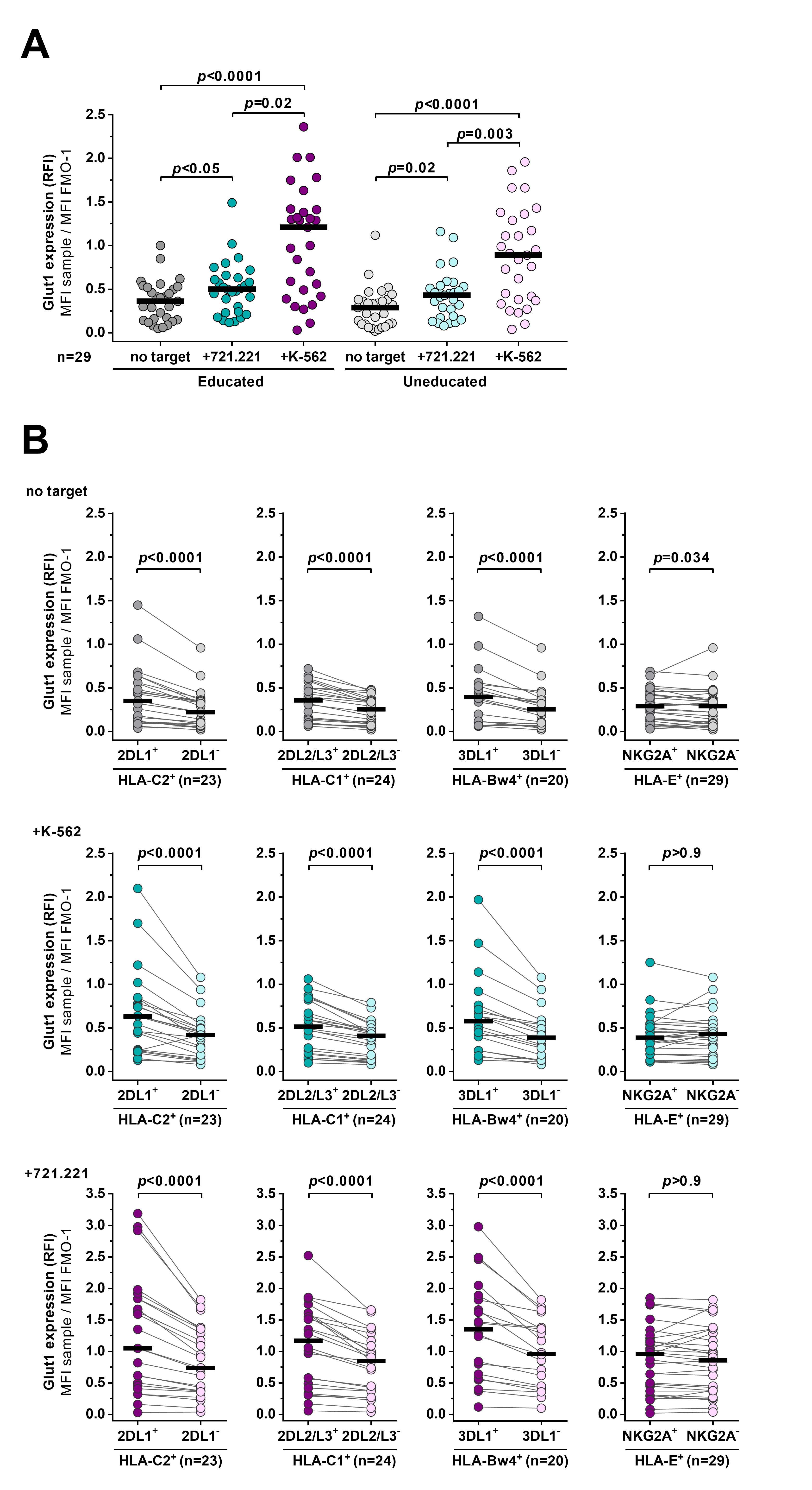
subsets

**Supplementary Figure 3 | Upregulation of Glut1 in educated and uneducated NK-cell subsets.**

Flow cytometric assessment of the glucose transporter Glut1 expression on NK cells with and without exposure to various target cells. Enriched primary NK cells from healthy donors were cultured for 4 h either in absence (gray) or in presence of 721.221 cells (cyan) or K‑562 cells (purple). **(A)** Comparison of Glut1 expression (RFI) within educated and uneducated NK cells. Statistical analysis: Friedman test, Dunn's multiple comparisons test. Black bars represent the median. **(B)** Comparison of Glut1 expression (RFI) between NK-cell subsets that are single positive for one of the investigated self-inhibitory receptors (2DL1, 2DL2/L3, 3DL1 or NKG2A) and subsets that lack all of the investigated self-inhibitory receptors in donors that express the cognate HLA class I molecule (Upper panel: no target, middle panel: +721.221, lower panel: +K-562). Statistical analysis: Wilcoxon matched‑pairs signed-rank test with subsequent Bonferroni correction. Black bars represent the median.

# Supplementary Tables

## Supplementary Table 1 | Key resource table

| **Reagent or resource** | **Conjugate** | **Clone** | **Source** | **Catalogue Number / Research Resource Identifiers (RRIDs)** |
| --- | --- | --- | --- | --- |
| **Antibodies** |  |  |  |  |
| α-human CD56 | BUV395 | NCAM16.2 | BD Bioscience | Cat#563554, RRID:AB_2687886 |
| α-human CD16 | BV785 | 3G8 | Biolegend, Inc. | Cat#302046, RRID:AB_2563803 |
| α-human CD107a | BV510 | H4A3 | Biolegend, Inc. | Cat#328632, RRID:AB_2562648 |
| α-human KIR3DL1 | BV421 | DX9 | Biolegend, Inc. | Cat#312714, RRID:AB_2561652 |
| α-human CD3 | PerCP-Cy5.5 | UCHT1 | Biolegend, Inc. | Cat#300430, RRID:AB_893299 |
| α-human CD14 | PerCP-Cy5.5 | HCD14 | Biolegend, Inc. | Cat#325622, RRID:AB_893250 |
| α-human CD19 | PerCP-Cy5.5 | HIB19 | Biolegend, Inc. | Cat#302230, RRID:AB_2073119 |
| α-human NKG2A | Pe-Cy7 | Z199 | Beckman Coulter | Cat#B10246, RRID:AB_2687887 |
| α-human KIR2DL2/L3/S2 | PE | DX27 | Biolegend, Inc. | Cat#312606, RRID:AB_2130554 |
| α-human KIR2DL1/S5 | APC | 143211 | R&D Systems | Cat#FAB1844A-100, RRID:AB_416855 |
| α-human KIR2DL1/S5 | FITC | 143211 | R&D Systems | Cat#FAB1844F-100, RRID:AB_2130402 |
| α-human KIR2DL3 | APC | 180701 | R&D Systems | Cat#FAB2014A-100, RRID:AB_416869 |
| α-human GLUT1 (SLC2A1) receptor binding protein (RBD) | eGFP |  | Metafora Biosystems | Cat#GLUT1-G100 |
| **Biological Samples** |  |  |  |  |
| Human peripheral blood mononuclear cells (PBMCs) |  |  | University medical center Hamburg-Eppendorf, Hamburg, Germany |  |
| **Chemicals** |  |  |  |  |
| LIVE/DEAD Fixable Blue Dead Cell Stain Kit, for UV excitation |  |  | Invitrogen | Cat#L23105 |
| Protein Transport Inhibitor (containing Monensin) / BD GolgiStop |  |  | BD Bioscience | Cat#554724 |
| 2-NBDG (2-deoxy-2-[(7-nitro-2,1,3-benzoxadiazol-4-yl)amino]-D-Glucose) |  |  | Biomol | Cay11046-1 |
| 2-DG (2-Deoxy-D-glucose) |  |  | Sigma-Aldrich | D3179-1G |
| RPMI 1640 Medium, no glucose |  |  | ThermoFisher | 11879020 |
| **Critical Commercial Assays** |  |  |  |  |
| EasySep Human NK Cell Enrichment Kit |  |  | Stemcell | Cat#19055 |
| Seahorse XF Glycolysis Stress Test Kit |  |  | Seahorse Bioscience/Agilent | Cat#103020-100 |
| **Cell lines** |  |  |  |  |
| LCL 721.221 |  |  | ATCC | ATCC Cat# CRL-1855, RRID:CVCL_6263 |
| K-562 |  |  | DSMZ | DSMZ Cat# ACC 10. RRID:CVCL_0004 |
| **Software and Algorithms** |  |  |  |  |
| FlowJo software v10.4.2 |  |  | Tree Star | <https://www.flowjo.com/>, RRID:SCR_008520 |
| Wave software, v 2.3.0.19 |  |  | Agilent | https://www.agilent.com/en/products/cell-analysis/cell-analysis-software |

## Supplementary table 2 | HLA class I genotyping

| **Number** | **Sample** **ID** | **HLA-A** | | **HLA-B** | | **Bw4/6^+^** | **HLA-C** | | **HLA-C Haplotype C1/C2** | **Glucose**  **Uptake** | **2-DG/**  **GfM** | **Seahorse Assay** | **Glut1 Assay** | **CD107a Assay** |
| --- | --- | --- | --- | --- | --- | --- | --- | --- | --- | --- | --- | --- | --- | --- |
|  |  | **A1** | **A2** | **B1** | **B2** |  | **C1** | **C2** |  |  |  |  |  |  |
| 1 | 180322_BC_A | 01:01 | 02:01 | 15:01 | 44:02 | 4 | 03:04 | 05:01 | 1/2 |  |  | + | + | + |
| 2 | 180322_BC_C | 01:01 | 23:01 | 37:01 | 44:03 | 4 | 04:01 | 06:02 | 2/2 |  |  |  | + | + |
| 3 | 180322_BC_D | 02:06 | 03:01 | 15:01 | 18:01 | 6 | 03:03 | 12:03 | 1/1 |  |  |  | + | + |
| 4 | 180316_BC_A | 03:01 | 25:01 | 35:01 | 44:02 | 4 | 04:01 | 07:04 | 1/2 |  |  | + | + | + |
| 5 | 180316_BC_B | 03:01 | 03:01 | 07:04 | 56:01 | 6 | 01:02 | 07:02 | 1/1 |  |  |  | + | + |
| 6 | 180316_BC_C | 01:01 | 36:01 | 08:01 | 15:03 | 6 | 04:01 | 07:01 | 1/2 |  |  |  | + | + |
| 7 | 180316_BC_D | 68:01 | 03:01 | 27:05 | 07:02 | 4 | 07:02 | 02:02 | 1/2 |  |  |  | + | + |
| 8 | 180308_BC_A | 01:02 | 29:02 | 27:05 | 49:01 | 4 | 02:02 | 07:01 | 1/2 |  |  |  | + | + |
| 9 | 180308_BC_B | 02:01 | 29:02 | 44:02 | 44:03 | 4 | 05:01 | 16:01 | 1/2 |  |  |  | + | + |
| 10 | 180308_BC_C | 01:01 | 24:02 | 08:01 | 40:02 | 4 | 02:02 | 07:01 | 1/2 |  |  |  | + | + |
| 11 | 180308_BC_D | 01:01 | 25:01 | 39:01 | 57:01 | 4 | 06:02 | 12:03 | 1/2 |  |  | + | + | + |
| 12 | 180201_BC_A | 02:01 | 31:01 | 15:01 | 51:01 | 4 | 03:04 | 15:02 | 1/2 |  |  | + | + | + |
| 13 | 180201_BC_B | 02:01 | 03:02 | 35:03 | 44:02 | 4 | 04:01 | 12:03 | 1/2 |  |  | + | + | + |
| 14 | 180201_BC_C | 02:01 | 03:01 | 35:01 | 57:01 | 4 | 04:01 | 06:02 | 2/2 |  |  |  | + | + |
| 15 | 180201_BC_D | 01:01 | 24:02 | 07:02 | 55:01 | 4 | 03:03 | 07:02 | 1/1 |  |  | + | + | + |
| 16 | 180110_BC_A | 02:01 | 11:01 | 08:01 | 44:02 | 4 | 05:01 | 07:01 | 1/2 |  |  |  | + | + |
| 17 | 180110_BC_B | 02:01 | 30:01 | 13:02 | 18:01 | 4 | 06:02 | 07:01 | 1/2 |  |  |  | + | + |
| 18 | 180110_BC_C | 01:01 | 02:01 | 07:02 | 52:01 | 4 | 07:02 | 12:02 | 1/1 |  |  |  | + | + |
| 19 | 180110_BC_D | 01:01 | 01:01 | 07:02 | 44:03 | 4 | 07:02 | 16:01 | 1/1 |  |  |  | + | + |
| 20 | 171129_BC_A | 01:01 | 23:01 | 37:01 | 44:03 | 4 | 04:01 | 06:02 | 2/2 |  |  | + | + | + |
| 21 | 171129_BC_B | 26:01 | 29:01 | 07:05 | 55:01 | 6 | 03:03 | 15:05 | 1/2 |  |  |  | + | + |
| 22 | 171129_BC_C | 02:01 | 02:01 | 51:01 | 51:01 | 4 | 14:02 | 16:02 | 1/2 |  |  |  | + | + |
| 23 | 171129_BC_D | 03:01 | 23:01 | 35:01 | 41:01 | 4 | 04:01 | 17:01 | 2/2 |  |  |  | + | + |
| 24 | 171109_BC_B | 02:01 | 29:02 | 35:01 | 44:02 | 4 | 03:04 | 05:01 | 1/2 |  |  |  | + | + |
| 25 | 171109_BC_C | 02:01 | 30:01 | 15:01 | 35:01 | 6 | 03:04 | 04:01 | 1/2 |  |  |  | + | + |
| 26 | 171109_BC_D | 01:01 | 32:01 | 40:02 | 57:01 | 4 | 02:02 | 06:02 | 2/2 |  |  |  | + | + |
| 27 | 171026_BC_B | 02:01 | 33:01 | 14:02 | 15:01 | 6 | 03:03 | 08:02 | 1/1 |  |  |  | + | + |
| 28 | 171026_BC_C | 01:01 | 68:01 | 08:01 | 35:01 | 6 | 04:01 | 07:01 | 1/2 |  |  |  | + | + |
| 29 | 171026_BC_D | 01:01 | 31:01 | 08:01 | 35:01 | 6 | 04:01 | 07:01 | 1/2 |  |  |  | + | + |
| 30 | 171011_BC_A | 02:01 | 11:01 | 08:01 | 44:02 | 4 | 05:01 | 07:01 | 1/2 |  |  | + |  | + |
| 31 | 171011_BC_B | 02:05 | 03:01 | 07:02 | 50:01 | 6 | 06:02 | 07:02 | 1/2 |  |  |  |  | + |
| 32 | 171011_BC_C | 01:01 | 03:01 | 07:05 | 08:01 | 6 | 07:01 | 15:05 | 1/2 |  |  |  |  | + |
| 33 | 171011_BC_D | 02:01 | 24:02 | 44:02 | 44:05 | 4 | 02:02 | 05:01 | 2/2 |  |  |  |  | + |
| 34 | 170816_BC_C | 02:01 | 24:02 | 15:01 | 44:05 | 4 | 02:02 | 03:03 | 1/2 |  |  | + |  | + |
| 35 | 170816_BC_D | 02:01 | 03:01 | 35:0 | 45:01 | 6 | 04:01 | 06:02 | 2/2 |  |  |  |  | + |
| 36 | 170810_BC_C | 01:01 | 24:02 | 44:02 | 57:01 | 4 | 05:01 | 06:02 | 2/2 |  |  | + |  | + |
| 37 | 170810_BC_E | 02:01 | 24:02 | 15:01 | 27:05 | 4 | 02:02 | 03:03 | 1/2 |  |  | + |  | + |
| 38 | 170727_BC_B | 03:01 | 25:01 | 35:01 | 44:02 | 4 | 04:01 | 07:04 | 1/2 |  |  | + |  | + |
| 39 | 170727_BC_C | 03:01 | 68:01 | 40:01 | 45:01 | 6 | 03:04 | 16:01 | 1/1 |  |  |  |  | + |
| 40 | 170727_BC_D | 02:06 | 32:01 | 35:01 | 37:01 | 4 | 03:03 | 06:02 | 1/2 |  |  | + |  | + |
| 41 | 170713_BC_D | 32:01 | 68:01 | 39:01 | 44:02 | 4 | 07:02 | 07:04 | 1/1 |  |  | + |  | + |
| 42 | 170706_BC_B | 02:01 | 03:01 | 15:01 | 40:01 | 6 | 03:04 | 03:04 | 1/1 |  |  | + |  | + |
| 43 | 170706_BC_C | 03:01 | 24:02 | 08:01 | 57:01 | 4 | 06:02 | 07:01 | 1/2 |  |  | + |  | + |
| 44 | 170629_BC_C | 01:01 | 02:01 | 08:01 | 58:01 | 4 | 03:02 | 07:01 | 1/1 |  |  | + |  | + |
| 45 | 170629_BC_D | 02:01 | 26:01 | 15:01 | 38:01 | 4 | 03:03 | 12:03 | 1/1 |  |  | + |  | + |
| 46 | HCHH102 | 02:01 | 66:01 | 15:01 | 15:17 | 4 | 03:04 | 17 | 1/2 | + | + |  |  |  |
| 47 | HCHH103 | 03:01 | 03:01 | 07:02 | 35:01 | 6 | 04:01 | 07:02 | 1/2 | + | + |  |  |  |
| 48 | HCHH160 | 02:01 | 02:01 | 13:02 | 40:01 | 4 | 03:04 | 06:02 | 1/2 | + | + |  |  |  |
| 49 | HCHH164 | 02:01 | 03:01 | 07:02 | 15:01 | 6 | 03:04 | 07:02 | 1/1 | + | + |  |  |  |
| 50 | HCHH181 | 02:01 | 02:01 | 08:01 | 13:02 | 4 | 06:02 | 07:01 | 1/2 | + | + |  |  |  |
| 51 | HCHH192 | 11:01 | 29:02 | 35:01 | 44:03 | 4 | 04:01 | 16:01 | 1/2 | + | + |  |  |  |
| **Total** |  |  |  |  |  |  |  |  |  | **6** | **6** | **18** | **29** | **45** |

Bw4^+^ donors were defined by carriage of HLA‑B alleles with a Bw4^+^ epitope and the HLA‑A alleles A-23:01, A‑24:02 and A‑32:01 (Foley *et al.*, Blood, 2008 doi:10.1182/blood-2008-01-132902). Bw6^+^ donors lack the above stated alleles.
